# Intercellular Interactions as Regulators of NETosis

**DOI:** 10.3389/fimmu.2016.00453

**Published:** 2016-11-14

**Authors:** Nayef M. Kazzaz, Gautam Sule, Jason S. Knight

**Affiliations:** ^1^Department of Internal Medicine, Division of Rheumatology, University of Michigan, Ann Arbor, MI, USA

**Keywords:** neutrophil extracellular traps, platelets, endothelium, selectins, integrins

## Abstract

Neutrophil extracellular traps (NETs) are chromatin-derived webs extruded from neutrophils in response to either infection or sterile stimulation with chemicals, cytokines, or microbial products. The vast majority of studies have characterized NET release (also called NETosis) in pure neutrophil cultures *in vitro*. The situation is surely more complex *in vivo* as neutrophils constantly sample not only pathogens and soluble mediators but also signals from cellular partners, including platelets and endothelial cells. This complexity is beginning to be explored by studies utilizing *in vitro* co-culture, as well as animal models of sepsis, infective endocarditis, lung injury, and thrombosis. Indeed, various selectins, integrins, and surface glycoproteins have been implicated in platelet–neutrophil interactions that promote NETosis, albeit with disparate results across studies. NETosis can also clearly be regulated by soluble mediators derived from platelets, such as eicosanoids, chemokines, and alarmins. Beyond platelets, the role of the endothelium in modulating NETosis is being increasingly revealed, with adhesive interactions likely priming neutrophils toward NETosis. The fact that the same selectins and surface glycoproteins may be expressed by both platelets and endothelial cells complicates the interpretation of *in vivo* data. In summary, we suggest in this review that the engagement of neutrophils with activated cellular partners provides an important *in vivo* signal or “hit” toward NETosis. Studies should, therefore, increasingly consider the triumvirate of neutrophils, platelets, and the endothelium when exploring NETosis, especially in disease states.

## Introduction

Neutrophil extracellular traps (NETs), first described in 2004, are released by neutrophils via an active process coined NETosis (1, 2). While first characterized for their role in combatting infectious organisms (1), these tangles of chromatin and antimicrobial proteins are now known to play a role in pathogenic autoimmunity and other sterile inflammatory states (3, 4). NETs may place organ systems at risk, including the vasculature (5–7), central nervous system (8), lungs (5), and kidneys (9, 10). Organ failure and thrombotic vessel occlusions are even possible (11–13). Neutrophils, as one of the first responders to inflammatory insults have long been known to interact with other cell types (especially platelets and endothelial cells) with implications for neutrophil recruitment, generation of reactive oxygen species (ROS), and phagocytosis. This cell-to-cell crosstalk may be mediated by either direct cell contact or soluble mediators. In this review, we will focus on the implications of crosstalk for NETosis. Relevant studies have characterized not only *in vitro* systems (typically with human cells) but also more complex murine models of disease. There is significant heterogeneity between studies, especially in terms of how NETosis is scored and the neutrophil pathways that are considered (which is probably not surprising as a canonical model of NETosis is still not established). Our goal is to highlight the similarities between studies and to point out the discrepancies that necessitate further research. Also, whenever possible, we will try to focus on the implications of these interactions for controlling infection and for regulating inflammation and end-organ damage.

## Platelet Function

Platelets are megakaryocyte-derived cell bodies that lack nuclei. They circulate in the bloodstream as well-established regulators of the hemostatic system (14). Platelets may be activated by the exposure of subendothelial matrix proteins, such as von Willebrand factor (vWF) and collagen, as might happen with mechanical vessel injury (15). Platelets recognize vWF via a glycoprotein receptor complex, glycoprotein Ib (GPIb)/IX/V (16), with the GPIb subunit playing a particularly key role (17). In parallel, collagen engages a different glycoprotein receptor, GPVI (18). Soluble plasma factors also activate platelets, including fibrinogen (via GPIIb/IIIa) (19) and thrombin (through protease-activated receptors or PARs) (20). When considering research studies, it is important to note that some studies may activate platelets with synthesized activators. An example is thrombin receptor activator peptide (TRAP), which acts as an agonist for all PARs (21), and the more specific TRAP-6, which binds specifically to PAR-1 (22).

These various activating signals lead to platelet aggregation and the release of copious amounts of preformed mediators from platelet granules, such as adenosine diphosphate (ADP) and thromboxane A_2_ (TXA_2_) – with the potential for potent local effects and feedforward into further platelet activation (14, 17). Platelet factor 4 (PF4, also known as C–X–C motif ligand 4) is another mediator released by platelets. In addition to functioning as a chemokine for cells, such as neutrophils, PF4 binds and neutralizes negatively charged cell surface glycosaminoglycans, such as heparan sulfate, dermatan sulfate, and chondroitin sulfate, thereby mediating several downstream effects, including platelet aggregation (23). Another soluble mediator that will be discussed in this article is high-mobility group box 1 (HMGB1), a protein “alarmin”/cytokine released by activated platelets (24). Finally, proteins such as P-selectin may be either released locally, or expressed on the platelet surface, thereby regulating the local environment (25, 26). For example, P-selectin has been implicated in platelet aggregation under pulsatile shear stress conditions (27).

While platelets clearly play a key role in stemming blood loss in the event of vessel injury, they also have well-established immunomodulatory properties, potentially acting as sentinels of infectious and inflammatory events (28, 29). In particular, the innate immune receptors toll-like receptor 2 (TLR2) and TLR4 (for Gram-positive and Gram-negative organisms, respectively) are expressed on the platelet surface (30, 31). Activation of these receptors may lead to release of platelet granules (32), PF4 upregulation (33), GPIIb/IIIa conformational changes (34), and ultimately feed forward to thrombin generation (30). Having said that, some studies have found less potent responses. For example, exposure of platelets to triacylated lipoproteins (like Pam3CSK4, a TLR2 agonist) and lipopolysaccharide (LPS, a TLR4 agonist) does not always lead to significant P-selectin release (35).

## Platelet–Neutrophil Interplay

Platelets interact directly with neutrophils and thereby alter neutrophil function (17). Examples of ligand/receptor pairs that mediate direct platelet/neutrophil interactions include P-selectin/P-selectin glycoprotein ligand 1 (PSGL-1) (36, 37), intercellular adhesion molecule 2 (ICAM-2)/lymphocyte function-associated antigen (LFA-1) (38), and GPIb/macrophage-1 antigen (Mac-1) (17, 39). These interactions clearly support platelet adhesion to leukocytes (40, 41) and, in some cases, have been shown to be of fundamental importance for recruitment of neutrophils to sites of inflammatory insult (40). Furthermore, beyond traditional direct interaction, some molecules (such as GPIIb/IIIa) may be transferred from platelets to neutrophils via microparticles (MP), thereby regulating neutrophil function (an example being nuclear factor kappa B activation) (42).

There is also a key role for platelet-released soluble mediators (ADP, TXA_2_, etc.) in both perpetuating platelet–neutrophil interplay and activating neutrophils. As an example, ADP (which would presumably be platelet-derived *in vivo*) induces platelet–neutrophil complexes through a mechanism that may be dependent upon P-selectin, but not PSGL-1 (41). TXA_2_ augments multiple neutrophil functions, including neutrophil adhesiveness (43), oxidative burst (44), and diapedesis (45). Platelet-derived HMGB1 can engage/activate neutrophil TLRs (46). Beyond TLRs, another well-recognized receptor for HMGB1 is the receptor for advanced glycation end products (RAGE), with engagement by HMGB1 leading to neutrophil recruitment and neutrophil-mediated tissue injury (47). PF4 interacts with neutrophil chondroitin sulfate (48) and (in the presence of co-stimulatory tumor necrosis factor alpha) mediates neutrophil granule release and surface adherence (49). PF4 has also been implicated in neutrophil chemotaxis (50). Neutrophil-activating peptide 2 (NAP-2) released from platelets can regulate neutrophil polarization and motility through CXCR1/2 (51). CCL5 (another chemokine released by platelets) may also play a role in neutrophil infiltration (52).

## Platelets and NETosis

Platelets are far-and-away the most studied cellular regulators of NETosis. Most model systems have pointed to platelet activation as the first step. This is followed by platelet–neutrophil crosstalk, and ultimately regulation of neutrophil effector function. Studies have employed numerous platelet activators, including LPS, Pam3CSK4, thrombin, collagen, ADP, and TRAP-6 (53–55). These different strategies for activation, beyond anything else, make it challenging to compare studies side-by-side (Table 1).

**Table 1:** **Selected *in vitro* studies of platelet-stimulated NETosis**.

Species	Platelet activator	Required mediator(s)	Not required	Reference
Human	LPS		P-selectin, Mac-1, GpIIb/IIIa	(54)
Mouse	LPS			(54)
Human	LPS	LFA-1		(56)
Human	*S. aureus* alpha toxin	hBD1		(57)
Human	TRAP	TXA_2_		(5)
Mouse		HMGB1 (via TLR4)	HMGB1 (via RAGE)	(58)
Human	Collagen, ADP, thrombin, TRAP-6	HMGB1	P-selectin, Mac-1, GpIIb/IIIa	(55)
Mouse	Collagen, ADP, thrombin, TRAP-6	HMGB1 (via RAGE)		(55)
Mouse	LPS		HMGB1	(55)
Human	TRAP, Pam3CSK4	TXA_2_, leukotriene B4, GPIb, vWF, LFA-1	P-selectin, GpIIb/IIIa	(53)
Mouse	Thrombin	P-selectin		(59)

*ADP, adenosine diphosphate; GPIb, glycoprotein Ib; GpIIb/IIIa, glycoprotein IIb/IIIa; hBD1, human beta-defensin-1; HMGB1, high-mobility group box 1; LFA-1 lymphocyte function-associated antigen 1; LPS, lipopolysaccharide; Mac-1, macrophage 1 antigen receptor; RAGE, receptor for advanced glycation end products; S. aureus, Staphylococcus aureus; TLR4, toll-like receptor 4; TRAP, thrombin receptor-activating peptide; TXA_2_, thromboxane A2; vWF, Von Willebrand factor*.

Regarding *in vitro* studies, platelet–neutrophil interactions have been assessed under static conditions (53, 57), and also with the introduction of shear stress (53–56). It is worth noting that the methodology for quantifying NETosis has varied markedly across studies. Examples include cell-free DNA quantification (53, 55), myeloperoxidase-deoxyribonucleic acid (MPO-DNA) ELISA (5, 55, 60, 61), neutrophil elastase-DNA ELISA (53), neutrophil elastase concentration (57), or direct visualization of NETs by fluorescence microscopy (54). Microscopy samples have been scored by quantifying percent surface area of Sytox green staining (detects extracellular DNA) (54, 58), histone H2Ax percentage surface area (56), or citrullinated histone H3-positive cells per field (62).

**Figure 1:**
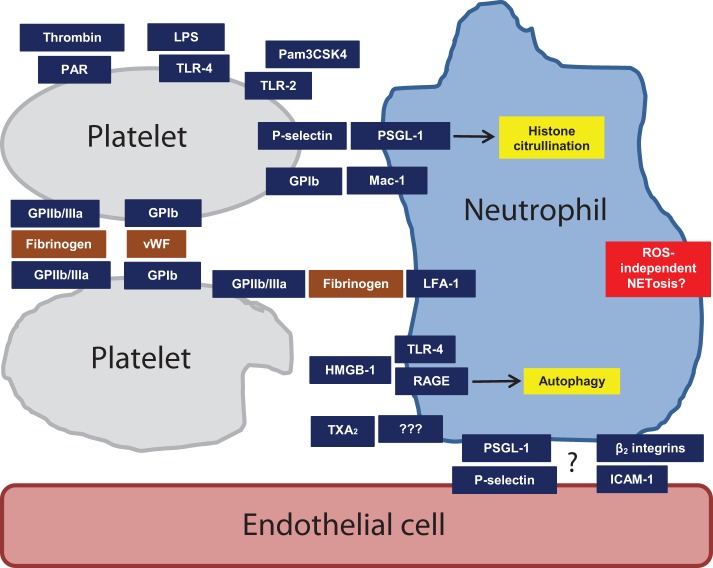
**Mechanisms of platelet activation and heterotypic intercellular interactions that may regulate NETosis**. Some are more speculative than others, as described in the text. Abbreviations: GPIb, glycoprotein 1b; GPIIb/IIIa, glycoprotein IIb/IIIa; HMGB1, high-mobility group box 1; LFA-1, lymphocyte function-associated antigen 1; LPS, lipopolysaccharide; Mac-1, macrophage-1 antigen; PAR, protease-activated receptor; PSGL-1, P-selectin glycoprotein ligand 1; RAGE, receptor for advanced glycation end products; TLR2, toll-like receptor 2; TLR4, toll-like receptor 4; TXA_2_, thromboxane A_2_; vWF, Von Willebrand factor.

We will first describe some notable *in vivo* studies in the field, which have focused on disease models (Table 2). We will then step through the various stages of platelet–neutrophil interplay, beginning with platelet activation and ending with NETosis (Figure 1).

**Table 2:** **Selected *in vivo* models of platelet-stimulated NETosis**.

Species	Model	Required mediator(s)	Reference
Mouse	Endotoxemia	LFA-1	(56)
Mouse	TRALI	GPIIb/IIIa	(5)
Mouse	ALI	HMGB1	(58)
Mouse	ALI	Mac-1, CXCL4/CCL5	(61)
Mouse	P-selectin overexpression	P-selectin	(59)
Rat	Endocarditis	P-selectin/PSGL-1	(63)
Mouse	IVC ligation	TXA_2_	(62)

*ALI, acute lung injury; CCL5, chemokine (C–C motif) ligand 5; CXCL4, (C–X–C motif) ligand 4; GpIIb/IIIa, glycoprotein IIb/IIIa; HMGB1, high-mobility group box 1; IVC, inferior vena cava; LFA-1, lymphocyte function-associated antigen 1; Mac-1, macrophage 1 antigen; PMA, phorbol 12-myristate 13-acetate; PSGL-1, P-selectin glycoprotein ligand 1; TRALI, transfusion-related acute lung injury; TXA_2_, thromboxane A2*.

### Notable *In Vivo* Models

One of the first studies to consider the impact of activated platelets on NETosis *in vivo* utilized a mouse model of endotoxemia (sepsis) induced by intravenous LPS (54, 56). The authors found that LPS triggers the recruitment of neutrophils to liver sinusoids, which then facilitate recruitment of platelets (54) – with platelet recruitment dependent upon neutrophil LFA-1 (56). Importantly, NETosis is only triggered after engagement by the activated platelets (which seem to have been primed by LPS acting through platelet TLR4). This functionality presumably plays a key role in bacterial sequester, but also places the host at risk for significant endothelial damage (54). The authors further mimic these data *in vitro*, demonstrating that stimulation of platelets through TLR4 enhances both platelet–neutrophil adhesion and NETosis, but without upregulating P-selectin expression or platelet aggregation (54).

Another notable study investigated platelet–neutrophil interplay in the context of transfusion-related acute lung injury (TRALI). TRALI was modeled by treating BALB/c wild-type mice with the combination of LPS and an anti-MHC I monoclonal antibody (5, 64). NETosis was quantified in the lungs by either intravital microscopy or postmortem histological examination (5). Lung NETosis was dependent upon platelet–neutrophil interplay as NETosis was significantly mitigated by inhibiting platelet activation with aspirin (an irreversible inhibitor of platelet TXA_2_ generation) or a GPIIb/IIIa inhibitor, tirofiban (5). *In vitro*, TRAP-activated platelets enhanced NETosis (5).

In a murine model of acute lung injury achieved with positive-pressure ventilation, platelet depletion led to depressed NETosis as measured in blood by MPO-DNA ELISA and in the lungs by microscopy (61). A critical role for Mac-1 was demonstrated with blocking antibodies and genetic knockout. By contrast, blocking LFA-1 did not suppress NETosis (61). Beyond integrin signaling, the authors argued that a second hit was also necessary for full neutrophil activation. Indeed, blocking platelet-derived CXCL4/CCL5 chemokine heterodimers reduced lung injury, while also explicitly mitigating NETosis in response to TRAP-activated platelets *in vitro* (61).

In a model of endocarditis, cultured bacteria from endocarditis patients were infused through carotid catheters into rats (63). By confocal microscopy, a platelet/bacteria layer was demonstrated inside the vegetation film, which was also intermixed with NETs (63). Furthermore, deoxyribonuclease (DNase, an enzyme that degrades DNA) proved to be an effective treatment (63). Platelets were deemed necessary for NETosis in this model, shown by inhibition with aspirin (63). Furthermore, NETosis was inhibited by P-selectin and PSGL-1 blocking antibodies (63).

In a final noteworthy study, the authors were interested in probing mechanisms by which aspirin might mitigate venous thrombosis (62). In a murine model of deep vein thrombosis (achieved by complete inferior vena cava ligation), both aspirin (which reduces the synthesis of TXA_2_ by platelets) and a selective thromboxane receptor antagonist reduced thrombus size. This was accompanied by a reduction in neutrophil infiltration, as well as deposition of both fibrin and NETs.

### Mediators of Direct Platelet–Neutrophil Interaction

#### P-Selectin/PSGL-1

If one considers *in vitro* studies with human neutrophils, then P-selectin has largely been judged dispensable for the ability of stimulated platelets to promote NETosis (53–55). In other species, the story may be different. For example, P-selectin has been implicated as required for thrombin-activated platelets to induce NETosis, as well as histone citrullination (a prerequisite for NETosis); this was demonstrated with cells isolated from knockout mice, and also by antibody-based inhibition (59). In the same study, mice overexpressing soluble P-selectin demonstrated higher neutrophil histone citrullination *in vivo*. Interestingly, P-selectin overexpression did not seem to regulate baseline NETosis, although accelerated NETosis could be unmasked in these mice with *ex vivo* stimulation (suggesting the neutrophils had been somehow primed by the overexpression) (59). Additionally, in the aforementioned rat model of infective endocarditis, platelet-induced NETosis was found to be dependent upon P-selectin/PSGL-1 as demonstrated by blocking antibodies (63).

What explains these discrepancies? One simple possibility is species difference (human versus mouse/rat). Another consideration is that P-selectin/PSGL-1 interactions may already be established when neutrophils are purified for *in vitro* studies, and so blocking antibodies may be less effective in this context (5, 65). As hinted above, the method of platelet stimulation must also be kept in mind, as there was no apparent role for platelet P-selectin in studies in which platelets were stimulated with LPS (54) or TRAP-6 (56), as compared to a positive role in a study using thrombin as the stimulus (59). As P-selectin may serve a priming role *in vivo* more so than as the primary stimulus (59), and as P-selectin is also well-known to be expressed on endothelial cells (66, 67), intravital studies that can probe these interactions in real time will be important in sorting this out going forward.

#### Neutrophil Mac-1

There is a suggestion that the β_2_ integrin Mac-1 is dispensable for platelet-induced NETosis based on *in vitro* studies with human neutrophils [with either TLR4 agonist (54) or TRAP-6 (5, 55) as the platelet stimulator]. By contrast, a study of acute lung injury demonstrated the requirement of Mac-1 for neutrophil-platelet aggregation as well as NETosis (61). Another interesting study recently revealed that neutrophil Mac-1 is required for crawling on the inflamed endothelium, a process that also requires PSGL-1, albeit without direct PSGL-1/endothelium contact (39). The authors discovered that PSGL-1 instead concentrates in a uropod, which projects into the bloodstream where it receives activating signals from platelets. These PSGL-1-mediated signals then regulate Mac-1 distribution and ultimately crawling (39). This study nicely highlights the potential complexity of platelet–neutrophil interplay *in vivo*, and how a comprehensive model of neutrophil effector functions (such as NETosis) may not be possible without considering both platelets and the endothelium.

#### Neutrophil LFA-1

The β_2_ integrin lymphocyte function-associated antigen 1 (LFA-1) is known to be the key receptor by which neutrophils interact with fibrinogen, an interaction that has been linked to an effective neutrophil oxidative burst (68). Beyond fibrinogen, platelet ICAM-2 may also interact with LFA-1 (38). *In vitro* studies with human platelets (activated with LPS, TRAP, or Pam3CSK4) have demonstrated that platelet–neutrophil interaction and resulting NETosis can be reversed with blockade of LFA-1 (53, 56), including under conditions of shear stress (56). Similarly, a mouse model of sepsis has supported a key role for LFA-1 in platelet-mediated NETosis, with either genetic deletion or blockade reducing NETosis in liver sinusoids (54, 56). However, in a different study focusing on murine neutrophils, TRAP-activated platelets signaled through neutrophil Mac-1, but not LFA-1, to induce NETosis (61). Differences in species, model, or culture conditions may have contributed to the discrepancies across studies.

#### Platelet GPIb

An *in vitro* study has suggested that GPIb (the classic receptor for vWF) is required for platelet-induced NETosis (53), although without a clear understanding of its counterpart on neutrophils. Interestingly, the authors also found that LPS-stimulated platelets increase expression and release of vWF, with blockade of vWF preventing platelet-induced NETosis (53). As GPIb can interact directly with neutrophils through Mac-1 (69, 70), and since vWF is also presented on the surface of endothelial cells, this pathway will need to be further dissected (including *in vivo*) before definitive conclusions can be drawn (71).

#### Platelet GPIIb/IIIa

In a mouse model of TRALI, blockade of GPIIb/IIIa (with tirofiban) reduced NETosis in lung tissue (5). This stands in contrast to *in vitro* human studies, which have not found a role for GPIIb/IIIa in platelet-induced NETosis (53–55). Interestingly, GPIIb/IIIa can be transferred from platelets to neutrophils through platelet-derived MP (42), an observation that could have implications for *in vitro* and *in vivo* discrepancies. It may also be that the key role of GPIIb/IIIa is to facilitate platelet–platelet or platelet–endothelial interactions (72–74), which would stand out in *in vivo* models, more so than the *in vitro* work.

### Soluble Mediators Released by Platelets

#### Eicosanoids

Platelets stimulated with Pam3CSK4 and TRAP (5, 53) may utilize TXA_2_ as a means of signaling to promote release of NETs (53). Given that there is no well-characterized receptor for TXA_2_ on neutrophils, mechanistic details remain to be determined.

#### Chemokines

PF4 (CXCL4) can play a role in regulating *in vitro* human NETosis, based on blocking experiments (53), and also direct stimulation of neutrophils with recombinant PF4 (53). *In vivo*, MKEY (a peptide inhibitor of CXCL4/CCL5 heterodimer formation) reduces NETosis in a model of acute lung injury (61).

#### Alarmins

Recombinant HMGB1 activates neutrophils to release NETs, dependent upon either neutrophil TLR4 (58) or neutrophil RAGE (55). Human beta defensin-1 (a microbicidal protein found in both neutrophils and platelets) is released by platelets exposed to *Staphylococcus aureus* alpha toxin, in a manner that then triggers NETosis (57).

### Neutrophil Signaling in Response to Platelets

It should be noted that neutrophil signaling has not been characterized in most models of platelet-induced NETosis. When Pam3CSK4, LPS, or TRAP were used to stimulate platelets, the resulting NETosis was found to be ROS independent (5, 53, 55). This is in contrast to *S. aureus* alpha toxin-activated platelets, which promote NETosis in a ROS-dependent manner (57). Platelet HMGB1 seems to leverage neutrophil autophagy to induce NETosis (55). Another study has demonstrated that ERK and PI3K are required for platelet-induced NETosis, when platelets were activated with Pam3CSK4, LPS, or arachidonic acid (53). At this point, the data are too limited to predict whether a consensus signaling pathway will emerge, although there are hints that ROS may not be a critically important factor in a critically important factor in platelet-induced NETosis.

## Endothelium–Neutrophil Interplay

Neutrophils develop in the bone marrow from myeloid precursors, reaching sites of infection or inflammation via the vasculature. This migration of neutrophils from the bloodstream to inflamed tissues is mediated by the interaction of adhesion molecules on the neutrophil surface with their respective ligands on the vascular endothelium. Details regarding this well-coordinated series of events arise from intravital microscopy studies in animals, as well as observations of patients with leukocyte adhesion deficiency (75). As an initial step, neutrophils leverage specific surface ligands in order to tether to P- and E-selectin molecules expressed on activated endothelial cells (selectin ligands potentially expressed on neutrophils include PSGL-1, E-selectin ligand 1, and CD44). Tethering of neutrophils is followed by their rolling along the endothelium (76–80). Rolling neutrophils develop membrane extensions at their rear end (tethers) and front (slings), which stabilize neutrophil rolling and allow the process to proceed despite the high shear stress of flowing blood (81). Subsequently, neutrophils firmly adhere to endothelial cells, mediated by the binding of neutrophil β_2_ integrins (LFA-1 and Mac-1) to endothelial ligands such as intracellular adhesion molecule 1 (ICAM-1) and ICAM-2 (76, 78, 79, 82). β_2_ integrins have two main states of activation: the first is an extended (but not open) form with low to intermediate affinity, and the second an extended and open form with high affinity (the form required for firm adhesion). Mechanisms and signaling pathways involved in these transitions have been delineated in great detail, and are reviewed elsewhere (82–84).

Rolling and adhesion may be followed by transmigration, when neutrophils pass between endothelial cells (paracellular) or through endothelial cells (transcellular). While many details remain to be determined, the paracellular process is more prevalent, occurring perhaps 90% of the time (76, 83, 85) and favored by neutrophils expressing Mac-1 (86, 87). By contrast, the transcellular route may be favored by increased endothelial expression of ICAM-1 (88) or by activation of endothelial cells by neutrophils through annexin A1 secretion (89). Beyond the above, adhesion molecules involved in the transmigration process include platelet endothelial cell adhesion molecule 1 (PECAM-1), CD99, ICAM-2, junctional adhesion molecules (JAMs), and cadherins (90). The roles of these adhesion molecules have primarily been demonstrated in mouse models wherein their deletion results in inhibition of transmigration and reduced accumulation of neutrophils in tissues (83, 85, 91).

Within inflamed tissues, neutrophils home via chemokine gradients. Interestingly, recent studies have demonstrated that neutrophils are able to undergo a “reverse transmigration” process such that tissue neutrophils may migrate back to the vascular lumen. Studies in mice have demonstrated that downregulation of JAM-C by neutrophil elastase plays a key role in the process (92). At present, the functional significance of reverse transmigration is not entirely clear. One idea is that the reverse transmigration has a significant downside, as it may contribute to dissemination of a local immune response into a systemic inflammatory phenomenon (93). Alternatively, it may play a role in dampening immune response as observed in zebrafish (94) and, we speculate patients with systemic inflammation (95).

Circulating neutrophils tend to be quiescent in nature, with their activation tightly linked to migration from circulation to tissue. Neutrophil activation can be thought of as a two-step process whereby exposure to one stimulus (priming) ensures a maximum response to a second. So, rolling and adhesion of neutrophils on the endothelium may initiate their activation, but full effector functions only become available to neutrophils once they encounter certain pro-inflammatory chemokines/cytokines or pathogen-derived ligands that can activate other receptors (G protein-coupled receptors and innate pattern-recognition receptors as classic examples). Neutrophils can then rapidly undergo degranulation, activation of their NADPH oxidase pathway for free radical generation, phagocytosis, and even NETosis (96–98). An example comes from studies of P-selectin overexpressing mice in which neutrophils seem to be sensitized to NETosis by excess P-selectin exposure, but do not actually release NETs unless confronted with a second stimulus (59).

## The Endothelium and NETosis

Netting neutrophils externalize not just chromatin but also a variety of antimicrobial peptides and proteases that target pathogens. Recent work has demonstrated that these mediators of host defense may also promote tissue damage (12). NETs induce endothelial cell death in a dose-dependent and partially DNA-independent manner (99). Rather than DNA, associated histones and to some extent myeloperoxidase may be most responsible for NET-mediated endothelial cytotoxicity (99). Another study demonstrated the externalization of matrix metalloproteinase-9 (MMP-9) and MMP-25 along with NETs. This externalized MMP-9 activates pro-MMP-2 produced by the endothelium, resulting in cytotoxicity and vessel dysfunction (100).

An interesting *in vitro* study investigated the implications of co-culture of activated endothelial cells with neutrophils (101). The result was not just increased NETosis by neutrophils, but also increased endothelial cell death (101). The death was attributable to increased IL-8 production by the endothelial cells themselves (101). One can imagine a scenario *in vivo* in which activated endothelial cells induce NETosis, followed by endothelial cytotoxicity and potentially the release of mediators that feed forward into more NETosis.

It should also be noted that although endothelial cells have not been the explicit focus of most NETs studies, they almost surely play a prominent role *in vivo*, either through direct regulation of neutrophil activity, or through modulation of other cellular elements, such as platelets (Figure 1). As an example, in the aforementioned sepsis model, liver sinusoids support neutrophil adhesion even in the absence of platelets, perhaps providing certain activating signals to the neutrophils that prime them for subsequent platelet capture (56). One might also point to the TRALI model (5). There, GPIIb/IIIa plays a key role in NETosis beyond anything that has been seen *in vitro* (53–55) – raising the question of whether additional synergistic signals may emanate from the endothelium *in vivo* (5). Finally, although studies focusing on platelet–neutrophil interactions *in vitro* have suggested contradictory roles for P-selectin (53–55), it is worth noting that P-selectin is also present on endothelial cells, which may help explain its more clear-cut role *in vivo* (59). We expect to see much more on this front in the coming years.

## Dendritic Cells

Dendritic cells (DCs) are best known for their role as professional antigen-presenting cells, bridging the gap between innate and adaptive immunity. In recent years, the intersection of neutrophils/NETosis and DCs has been increasingly considered. First, neutrophils are well established to play a role in the recruitment of DCs to sites of inflammation, and promote maturation of DCs via secretion of a variety of soluble mediators, such as CCL3, CCL4, CCL5 (RANTES), CCL20, tumor necrosis factor α, α-defensins, and cathelicidins (102–106). At the same time, *in vivo* immunization studies have demonstrated that neutrophils can dampen immune responses by competing for antigen with DCs and limiting contact between T cells and DCs (107). So, at least in some contexts, vaccination responses may improve with temporary depletion of neutrophils. In other contexts, NETs seem to do the opposite, quite specifically transferring antigens to DCs, and thereby initiating autoimmune disorders, such as small vessel vasculitis (108).

With further implications for autoimmunity and sterile inflammation, NETs activate plasmacytoid DCs in lupus and atherosclerosis via TLR9. Activated plasmacytoid DCs produce interferons, which in turn prime neutrophils for more NETosis (thereby setting up a positive feedback loop) (109, 110).

Again pointing to different roles in different contexts, DCs may sometimes downregulate NETosis. This has been described in the specific context of human immunodeficiency virus (HIV), which acts through CD209 on DCs to produce interleukin 10 (IL-10). IL-10 then inhibits HIV/TLR7-mediated NETosis (111). Demonstrating at least some specificity, PMA-induced NETosis is not suppressed by IL-10 (111).

## Microparticles

MP are small, cell membrane-derived vesicles (112). MP from endothelial cells (113, 114), platelets (115), and red blood cells (116) have all been implicated in activating neutrophils. Furthermore, both platelet-derived (115) and red blood cell-derived (116) MP induce Mac-1 expression on neutrophils and stimulate neutrophil phagocytic activity (115, 116). The role of MP in promoting NETosis was also demonstrated in a paper focusing on preeclampsia, in which placenta syncytiotrophoblast-derived MP seem to promote NETosis (117). In inflammatory bowel disease, MP also appear to activate NETosis (118).

## Clearance of NETs

While NETs play a critical role in host defense, excessive formation or persistence of NETs may lead to adverse effects. Thus, clearance of NETs is an important physiological process that helps minimize excessive presentation of both toxic products and potential self-antigens. Degradation of NETs by serum DNase is one mechanism by which NETs are cleared, with impairment of this process leading to a lupus-like syndrome in mice (119). Interestingly, inadequate DNase activity has also been detected in the blood of patients with both lupus (119–121) and autoimmune vasculitis (122). Beyond the enzymatic activity of DNase, macrophages also play a role in the clearance of NETs. DNase processing of NETs prepares them for engulfment by macrophages, with the process further facilitated by the opsonization of NETs by complement C1q (123). Though this process was initially thought to be immunologically silent, recent *in vitro* studies have demonstrated a potentially complicated response that depends upon macrophage polarization (124). The authors show that M2 macrophages induce a pro-inflammatory response when exposed to NETs (including the release of a variety of pro-inflammatory cytokines/chemokines). By contrast, M1 macrophages initially undergo cell death that leads to their own nuclear decondensation and DNA release. Interestingly, over time, M1 macrophages then degrade this macrophage-derived DNA in a caspase-activated DNase-dependent manner (124). The full implications of this interplay remain unclear *in vivo* (and in disease states) and will hopefully be elucidated by future studies.

## Future Directions

This is a field in which much remains to be defined, as is especially highlighted by the various studies of platelet-induced NETosis. Studies in different systems and by different investigators have revealed surprisingly little mechanistic consensus, which probably points to an involvement of multiple pathways, thereby allowing certain aspects to be revealed by different groups. An obvious barrier is that platelet activation is achieved through different methodology in each study. It would be very interesting to see one group (or preferable a number of groups) take a systematic approach to this question, asking how the method of stimulation influences the specifics of platelet–neutrophil crosstalk. Given the highly regulated crosstalk that exists between the endothelium and neutrophils, endothelial cells surely play an important role in regulating NETosis *in vivo* – although relatively few studies have specifically probed that role. Studies should, therefore, increasingly consider the triumvirate of neutrophils, platelets, and the endothelium when exploring NETosis, especially in disease states.

## Author Contributions

NK and GS wrote the review. JK edited and approved the final version for publication.

## Conflict of Interest Statement

The authors declare that the research was conducted in the absence of any commercial or financial relationships that could be construed as a potential conflict of interest.
